# Upgraded imaging capabilities at the BAMline (BESSY II)

**DOI:** 10.1107/S1600577522007342

**Published:** 2022-08-17

**Authors:** H. Markötter, M. Sintschuk, R. Britzke, S. Dayani, G. Bruno

**Affiliations:** a Bundesanstalt für Materialforschung und -Prüfung, Unter den Eichen 87, 12205 Berlin, Germany; b University of Potsdam, Karl-Liebknecht-Strasse 24/25, 14476 Potsdam-Golm, Germany; Bhabha Atomic Research Centre, India

**Keywords:** synchrotron radiation, computed tomography, double-multilayer monochromators, pink beams, X-ray optics

## Abstract

The BAMline at the BESSY II synchrotron X-ray source has enabled research for more than 20 years in widely spread research fields such as materials science, biology, cultural heritage and medicine. As a nondestructive characterization method, synchrotron X-ray imaging, especially tomography, plays a particularly important role in structural characterization. A recent upgrade of key equipment of the BAMline widens its imaging capabilities: shorter scan acquisition times are now possible, *in situ* and *operando* studies can now be routinely performed, and different energy spectra can easily be set up. In fact, the upgraded double-multilayer monochromator brings full flexibility by yielding different energy spectra to optimize flux and energy resolution as desired. The upgraded detector (based on an sCMOS camera) also allows exploiting the higher flux with reduced readout times. Furthermore, an installed slip ring allows the sample stage to continuously rotate. The latter feature enables tomographic observation of processes occurring in the time scale of a few seconds.

## Abstract 2

A recent upgrade of key equipment of the BAMline widens its imaging capabilities: shorter scan acquisition times are now possible, *in situ* and *operando* studies can now be routinely performed, and different energy spectra can easily be set up.

## 1. Introduction

Synchrotron X-ray imaging (SXI) is applied at synchrotron sources around the world in a broad range of scientific fields, such as materials science, biology, cultural heritage, geoscience and medicine (Arhatari *et al.*, 2021 ▸; Olbinado *et al.*, 2018 ▸; Schmelzle *et al.*, 2021 ▸; Gao *et al.*, 2021 ▸; Nogueira *et al.*, 2017 ▸; Longo *et al.*, 2007 ▸; Chen *et al.*, 2020 ▸; Kandula *et al.*, 2022 ▸; Rau *et al.*, 2010 ▸). Relevant to BAM (Bundesanstalt für Material­forschung und -Prüfung) competencies, SXI proves suitable for nondestructive studies of materials and (small) components (Mayo *et al.*, 2012 ▸; Heenan *et al.*, 2019 ▸; Lin *et al.*, 2017 ▸). Besides radiography of dynamic processes (Wang *et al.*, 2020 ▸; Hu *et al.*, 2018 ▸), use of special imaging setups such as X-ray refraction radiography (Nellesen *et al.*, 2018 ▸; Cabeza *et al.*, 2018 ▸; Kupsch *et al.*, 2017 ▸), phase contrast (Bronnikov, 2002 ▸) and ptychography (Rodenburg & Maiden, 2019 ▸), SXI is used for 3D characterization by means of classic absorption-based tomography (SXCT) (Bonse & Busch, 1996 ▸; Liu *et al.*, 2016 ▸). The BAMline is a hard X-ray beamline at BESSY II, HZB, Berlin, Germany, used for imaging in materials science. It was commissioned in 2000 (Görner *et al.*, 2001 ▸) and is used for spectroscopic techniques (Riesemeier *et al.*, 2005 ▸; Vasilescu *et al.*, 2010 ▸; Buzanich *et al.*, 2016 ▸), and also for X-ray computed tomography (XCT) (Rack *et al.*, 2008 ▸). Since then, improvements have been focused on the detection capabilities of the CCD camera, aiming towards a higher pixel resolution. A more extensive recent upgrade, mainly regarding the double-multilayer monochromator (DMM), and indeed the whole detection chain, was carried out. The details of the upgrade and the opportunities enabled by these improvements are described in the present article.

## 2. Beamline layout

A 7 T wavelength shifter (WLS) with a critical energy of 13.5 keV is used as an insertion device. This allows the coverage of an energy range roughly between 8 and 60 keV. A DMM and a double-crystal monochromator (DCM) are installed ∼20 and 27 m downstream of the WLS, respectively. They can be used individually or in combination. Slit systems are installed at three positions; they are used to block scattered X-rays and to shape the beam as a rectangle according to the rectangular detector area. A filter system including Be, Al and Cu foils is used to suppress the lower-energy X-rays.

Recently, the DMM has been upgraded to now offer three beam configurations. This is realized by having three stripes with different (multilayer) coatings. Two of them are coated with multilayers selecting only the energy fulfilling the Bragg-like law, *i.e.* the wave propagation within the multilayer fulfills the Bragg condition. The last stripe is coated with a single layer used under total reflection, offering a wider spectrum. These options are described in the *DMM upgrade* section below. They offer energy resolutions Δ*E*/*E* down to ∼1.5%. The DCM’s energy resolution is ∼0.01%. Therefore, the DCM can be used in cases for which a very high energy resolution is needed, such as 2D X-ray absorption spectroscopy imaging (Arlt *et al.*, 2013 ▸) or refraction-enhanced imaging (SXRR, SXRCT) (Müller *et al.*, 2009 ▸; Nellesen *et al.*, 2018 ▸).

The sample manipulator offers the necessary positioning (*x*, *y*, *z*; PI miCos) and tilting (around the horizontal axes; HUBER Diffraktionstechnik GmbH), as well as an air-bearing rotation stage (around the vertical axis; PI miCos), which is equipped with piezo-driven stages (Micronix USA) to bring the sample onto the rotation axis. So far, the detector has been based on a CCD camera, optimized for a high signal-to-noise ratio, but with readout times in the range of seconds (for each frame). This large readout time has prevented any rapid sequential imaging experiment. Therefore, the detector has been adapted to a higher beam flux by upgrading to an sCMOS camera, which is described in the *Detector system upgrade* section.

## 3. DMM upgrade

Besides the filters, the monochromators are the most important optical elements for beam conditioning. The WLS delivers a wide spectrum. Therefore, the X-ray energy spectrum provided by the monochromators determines the kind of experiment one can perform and the interpretation of the results. Thus, knowledge about the spectrum is paramount; even more, flexibility in shaping the spectrum is a requirement. As mentioned above, the BAMline is equipped with a DMM and a DCM. While the DCM delivers a high energy resolution, but only a limited amount of flux, the DMM is most suitable for high-spatial-resolution XCT scans in a reasonable amount of time.

**Figure 1:**
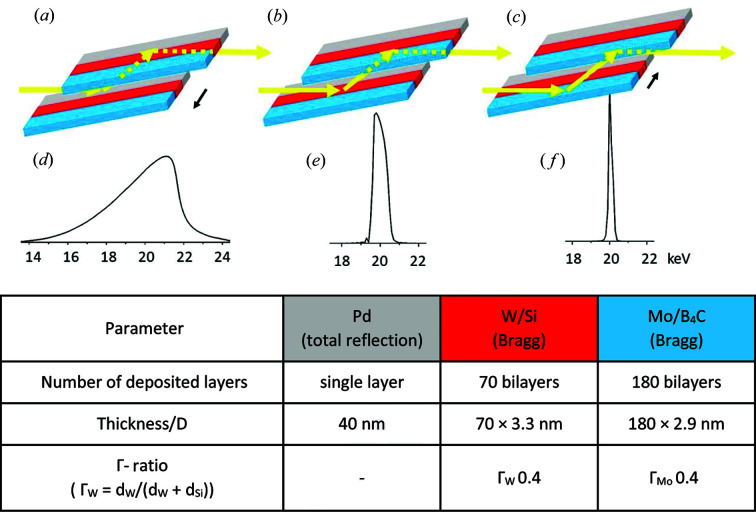
The DMM in three-stripe design; lateral movement of the DMM selects one of the stripes. (*a*) Pd layer, (*b*) W/Si multilayer and (*c*) Mo/B_4_C multilayer. These allow different spectral characteristics to be obtained. Calculated example spectra for 20 keV are given in (*d*)–(*f*) (the *y* axis is in a.u.); layer parameters are reported in the table.

The DMM consists of two Si substrates, 320 and 380 mm in length, and each 80 mm wide. The horizontal beam size of up to ∼20 mm allows an efficient use of the width of the substrates, since the substrates have three stripes with different coatings, each with different characteristics. These are optimized for different spectral resolutions. The coating was conducted by AXO (Dresden, Germany) on substrates produced by Carl Zeiss SMT (Germany). In Fig. 1 ▸ the pair of multilayer mirrors is shown in three different positions. Lateral movement (in the horizontal direction) of the whole DMM vacuum chamber allows one to select only one of the stripes. In Fig. 1 ▸(*a*) the beam hits the stripe with a single palladium (Pd) layer, while in Figs. 1 ▸(*b*) and 1 ▸(*c*) tungsten silicon (W/Si) and molybdenum boron carbide (Mo/B_4_C) multilayer stripes are exposed, respectively.

The single Pd layer is used in total reflection mode, while for the W/Si and the Mo/B_4_C multilayer stripes Bragg-like diffraction is used. Example calculated spectra are shown in Figs. 1 ▸(*d*) to 1 ▸(*f*) to emphasize the difference in the possible spectral resolutions [calculations were conducted with the inhouse graphical user interface *BAMline-Helper* based on the Python *xrt* package (Klementiev & Chernikov, 2014 ▸)]. The spectrum reflected by the Pd layer depends on the incident angle θ [Fig. 1 ▸(*d*) shows the spectrum calculated with θ = 0.17°]. Since the refractive index decreases with higher energies, smaller incident angles must be set to allow total reflection. Hence, smaller angles shift the high end of the spectrum to higher energies and larger angles to smaller energies. The low end of the spectrum is tailored by choosing the filters available (at the beamline: Be, Al, Cu). Five examples are given in Table 1 ▸. Since the Pd layer delivers a nearly white spectrum, the spectrum is also denoted as ‘pink beam’. There is a geometrical constraint limiting the vertical beam size: the grazing-incidence angle at which the beam hits the DMM mirrors. Therefore, depending on the DMM angle θ (and hence beam energy), the vertical beam size at the experiment is limited to ∼1 mm. A list of available energies and vertical beam sizes is given in Table 1 ▸.

The other two coatings are used under Bragg-like conditions. The W/Si coating consists of 70 bilayers, resulting in an energy resolution of ∼4%. In fact, the resolution depends on the number of layers contributing to the diffraction, *i.e.* the penetration depth and the angle θ. In Table 1 ▸, calculated values for the full width at half-maximum (FWHM and Δ*E*/*E*) of the spectra as well as the available vertical beam size depending on the beam energy are given. The DMM area coated with Mo/B_4_C consists of 180 bilayers and monochromatizes the beam to ∼1.5% Δ*E*/*E*. Thus, if elements known from the sample have absorption edges in a close range, and these are to be separated, then the Mo/B_4_C option is preferable. In all other cases, the W/Si coating is superior for CT experiments, providing the highest flux with sufficient energy resolution. This is also an improvement in relation to the previously used DMM [W/Si, 150 bilayers, ∼1.7% Δ*E*/*E* (Rack *et al.*, 2008 ▸)]. A more in-depth article describing the technical details of the beamline optics (DMM, DCM) is in preparation.

## 4. Detector-system upgrade

To fully exploit the easy access to different X-ray spectra provided by the DMM, including the pink-beam option, the detector system was also upgraded. The employed new microscope is optimized for white X-ray beams and is combined with an sCMOS-based camera with 2560 × 2160 pixels. The microscope is equipped with CdWO_4_ scintillators.

### 4.1. White-beam microscope

**Figure 2:**
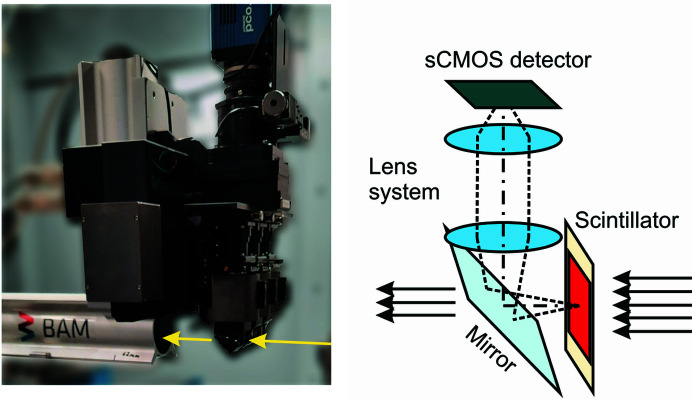
Photograph and sketch of the detector setup in white-beam configuration.

The microscope system was manufactured by Optique-Peter (Lentilly, France) and is designed in a white-beam configuration to meet the requirements of imaging experiments using high X-ray fluxes. In comparison with the design for monochromatic beams, the first lens is not located directly behind the scintillator but above the mirror that folds the optical path of the visible spectrum upwards to the camera (see sketch in Fig. 2 ▸, right). This keeps the lens away from any transmitted X-rays and avoids degradation of the optics (Douissard *et al.*, 2012 ▸). In the previous design (for monochromatic beams), the lens sitting right behind the scintillator represented inconvenient reducing of the lens transparency in the visible-light range, during and after experiments using high energies. The darkening depends on the lens type chosen. In fact, in such experiments, part of the beam transmitted by the sample traverses the scintillator and hits the lens.

A horizontal motorized linear stage allows switching among three detector heads, each of them carrying different optics and hence resolutions/fields of view. There are four available optics, which allow covering sample sizes up to 9.2 mm width or pixel sizes down to 0.36 µm. The objectives (from Mitutoyo) were reworked by Optique-Peter and equipped with a front radiation-protective window or lens. The choice of lenses with their numerical aperture (NA), fields of view and the corresponding pixel sizes is given in Table 2 ▸. As each head carries its own scintillator, the scintillator thicknesses are adapted to each magnification. Each detector head has its own motorized focus stage.

### 4.2. sCMOS camera

The new camera model is a pco.edge 5.5 with 2560 columns and 2160 rows, and hence 5.5 megapixels with a native pixel size of 6.5 µm. Since the sensor is based on sCMOS technology, readout times of a few milliseconds can be achieved. This allows reading of 100 full frames per second (fps). However, by vertically reducing the region of interest or by binning multiple pixels, even higher frequencies are possible. Especially when using the pink-beam option, only a limited vertical beam size is available (see Table 1 ▸); consequently, the readout sensor region can be efficiently cropped. The first XCT experiments with a pink beam indicated that 100 fps are sufficient to avoid saturation of the signal. However, for dynamic radiography (radioscopy), one could further reduce the exposure time to reach more than 1000 fps. This would enable recording of fast processes, though at the price of a lower spatial resolution and/or signal-to-noise ratio.

### 4.3. Scintillators

The used CdWO_4_ scintillators were produced by Saint-Gobain India Private Limited – Crystals. Two different sizes and thicknesses are used, both are placed on a 600 µm SiO_2_ substrate (Suprasil 2 Grade A). One set of scintillators is 10 mm × 10 mm large and 150 µm thick, while the other set is 6 mm × 6 mm large and 60 µm thick. The proper scintillator/lens combination (see Table 2 ▸) adapts the detector to the desired resolution and flux. The CdWO_4_ emission spectrum has a peak wavelength of ∼475 nm (Martin & Koch, 2006 ▸), which fits well with the sensitivity curve of the sCMOS sensor. With a CdWO_4_ decay time of ∼14 ms, frame rates of 100 fps are still reasonable, only leading to smearing between subsequent projections, hence reducing the resolution (up to 50 fps shown in Fig. 5). With a density of 7.9 g cm^−3^, this scintillator type strongly absorbs X-rays, *e.g.* a 60 µm CdWO_4_ is still absorbing 39% of the impinging beam at 40 keV. Below 40 keV (or with a thicker screen), an even higher fraction of X-rays is converted into visible light.

### 4.4. Resolution

**Figure 3:**
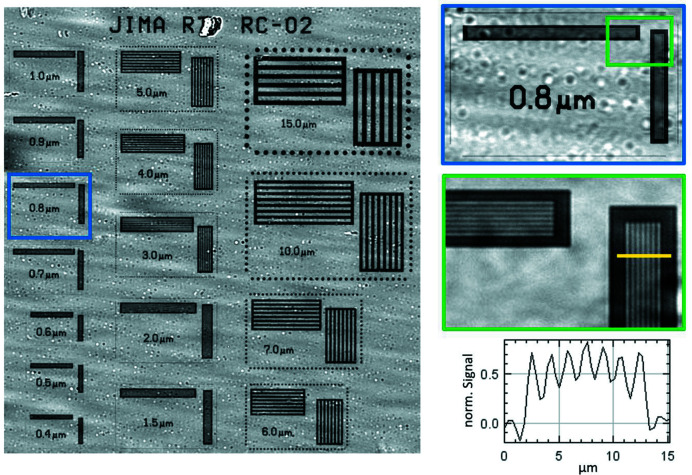
JIMA resolution target; enlarged view of 0.8 µm lines; detail and a plot through lines. The circles in the blue insert are normalization artefacts due to beam instabilities.

Fig. 3 ▸ shows the resolution achievable using the 20× lens, offering the highest available magnification. Radiography of a JIMA target was performed by averaging five acquisitions (plus five flat fields) of 1.5 s exposure time each. The test was conducted with a sample–detector distance of ∼12 mm at a beam energy of 11 keV using the W/Si DMM and the 60 µm CdWO_4_ scintillator. The 0.8 µm lines of the grid are still clearly visible. For the line widths of 0.9 and 0.8 µm, object-normalized modulations of 43 and 18% are obtained, respectively. For SXI in parallel-beam geometry, these are expected values considering an effective pixel size of 0.36 µm. In fact, with the NA of the 20× lens of 0.41 and the emission wavelength of CdWO_4_, the Rayleigh criterion would predict a theoretical resolution limit of 0.71 µm. At 11 keV, the X-rays are converted into visible light within a few micrometres. At higher beam energies, the X-ray conversion will also take place in deeper layers of the scintillator and thus negatively affect the achievable resolution.

## 5. Slip ring

**Figure 4:**
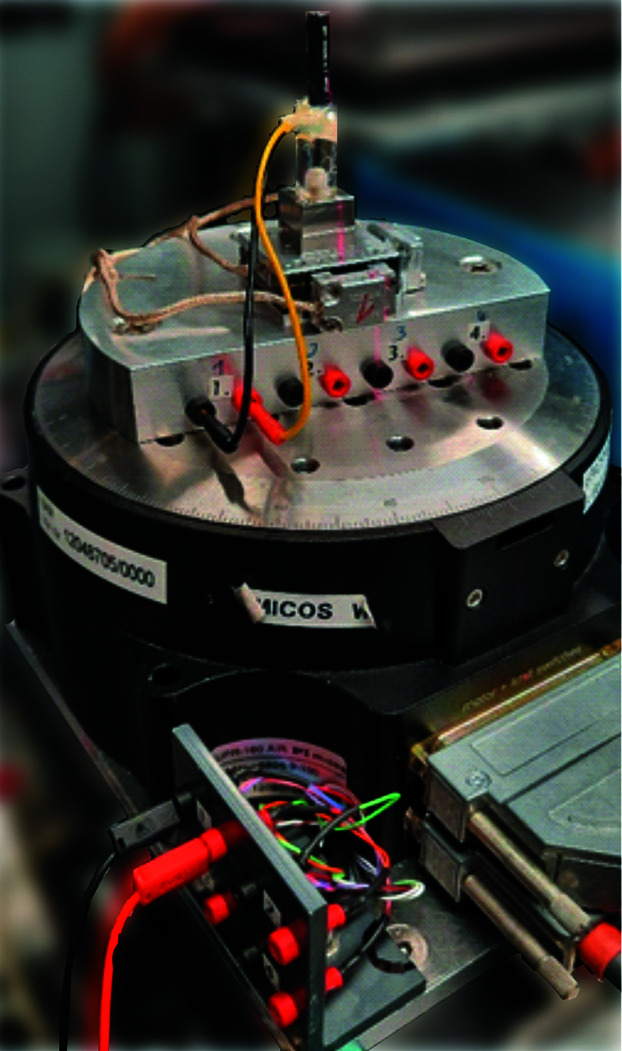
A slip ring offering eight channels (each with currents of up to 1 A) to facilitate cable management during rotation of the sample stage.

The reduction of scan times, provided by the new DMM and detector, enables extensively broader *in situ* and *operando* studies with better time resolution. Often, however, cabling of the sample positioning stage and of the *in situ* or *operando* cell prevents an efficient use of such shorter scan times, as such cables do not allow continuous rotations. A back-and-forth angular scanning strategy, each covering 180° plus a short angular range to allow for slowing down and speeding up, is commonly necessary to circumvent the problem of cabling. Moreover, additional data-analysis effort is necessary for registering the reconstructed volumes. To solve the problem, a slip ring has been installed, placed within the aperture of the rotation stage, to feed cables through the rotation stage without winding them. A model with a total number of 36 channels, each allowing currents of up to 1 A, was chosen (Senring M220A-36). Of these, 24 channels are used for the piezoelectric driven sample position stages (including the readback signal), and eight channels are available for the sample environment on two panels with 2 mm banana sockets (see Fig. 4 ▸). This is very convenient for *in situ* and *operando* studies (see below).

## 6. Scan options

**Figure 5:**
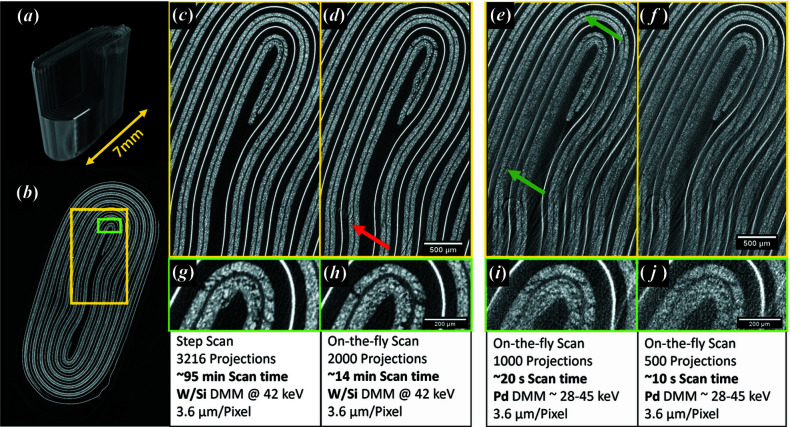
Scan options shown in the example of an Li pouch cell with 7 mm width. (*a*) 3D view, (*b*) horizontal slice of a standard scan strategy and reconstruction (see main text), (*c*) monochromatic beam in step-scan mode; (*d*) monochromatic beam in on-the-fly scan mode; polychromatic (pink) beam in on-the-fly scan mode acquired for (*e*) 20 s and (*f*) 10 s; (*g*)–(*j*) enlarged detail of (*c*)–(*f*).

The upgraded equipment allows new possibilities as both XCT scanning strategy and energy spectrum are concerned. With the options in energy resolution and X-ray flux, the scanning times range from more than one hour (previously commonly available at the BAMline) down to a few seconds. Fig. 5 ▸ gives a few examples showing reconstructions of SXCT data of a 7 mm-wide Li battery [reconstructed with the Python package *TomoPy* using the Gridrec algorithm and Shepp–Logan filter (Gürsoy *et al.*, 2014 ▸; Marone & Stampanoni, 2012 ▸)]. Shown is a battery’s jelly roll consisting of a metal oxide cathode on an aluminium current collector and a graphite-based anode dispersed on a copper current collector separated by a polymer-based separator. The XCT scans were carried out at an energy of 42 keV and a pixel size of 3.6 µm. In Fig. 5 ▸(*a*) a 3D view and in Fig. 5 ▸(*b*) a complete horizontal slice are shown. Figs. 5 ▸(*c*)–5 ▸(*f*) show magnified areas while Figs. 5 ▸(*g*)–5 ▸(*j*) show enlarged details of such areas. Particle segmentation of the cathode material, investigating the separator’s integrity and tracking the anode’s morphology require clean reconstructions with a high signal-to-noise ratio. However, fast scans (with lower signal-to-noise ratio) are required when monitoring *operando* degradation, which leads to general morphology change such as jelly-roll deformation or volume change due to gas-evolution reactions.

Data in Figs. 5 ▸(*c*)–5 ▸(*f*) were acquired using different strategies: in Fig. 5 ▸(*c*) [and 5 ▸(*g*)] the battery was scanned in step-scan mode with 3216 projections, meaning that no movement during the exposure was carried out, the rotation and positioning stages were moved only subsequently, and after completion of the movement the next exposure was started. Due to the motor movement and to the unavoidable communication times, the scan time was inefficiently used. However, this scanning strategy allows full flexibility with respect to the order, the position and to the angle at which the projections are recorded. In this way, interlaced scanning strategies and ring-artefact suppression can be implemented, thereby allowing further improvement of the reconstruction results (Zhu *et al.*, 2013 ▸). Indeed, the red arrow in Fig. 5 ▸(*d*) depicts a remnant of a ring artefact, which is not present in Fig. 5 ▸(*c*). The reconstruction in Fig. 5 ▸(*c*) is based on a tomographic scan with 16 sequences, each covering 180° with 201 projections. The sequences were started with different starting angles, interlaced with each other. In between the sequences, the axis was laterally moved, which distributes ring artefacts onto several tracks. Therefore, the ring artefacts vanish.

In Fig. 5 ▸(*d*) [and 5 ▸(*h*)] the battery was scanned ‘on the fly’: the sample was slowly rotating during consecutive radiographic exposures. This makes the most effective use of beam time, as nearly the whole scan time is used for the exposures, leaving aside the movement to flat-field position and the exposure of the flat fields. While the signal-to-noise ratio is not much lower than in the step-scan approach [compare Figs. 5 ▸(*g*) and 5 ▸(*h*)], the scan time is drastically cut down in the case of the on-the-fly scan procedure.

In the case when the signal-to-noise ratio is less important than the temporal resolution, scan times can be further cut down by reducing the number of projections as well as by using a pink beam. The higher flux available in the pink-beam mode easily brings the scan times well below 1 min. This is shown in Figs. 5 ▸(*e*) and 5 ▸(*f*), which show reconstructions of full XCT scans acquired with 20 s and 10 s scan times, respectively. The Pd layer delivered a pink beam with an energy range of roughly 28 to 45 keV. Without pre-processing, the reconstructed volumes suffer from beam hardening, as can be seen at the reconstruction of the cathode marked by the green arrows in Fig. 5 ▸(*e*). Despite such artefacts, the reconstruction of the copper current collector (the bright lines) can still be used to study, for example, a volume expansion or a deformation of the pouch under load or during penetration tests. An even further increase of the repetition rate could be realized with pixel binning to allow faster readout and to cope with the lower number of photons per exposure.

## 7. Summary and conclusions

We have described the salient features and the key elements of the recently upgraded BAMline (BESSY II, HZB, Berlin, Germany). Such new features are a DMM, a more robust optics and camera system, and a slip ring built into the turntable. Such features have been designed to extend the XCT capabilities, particularly for *in situ* and *operando* imaging experiments. In fact, they allow drastic reduction of the scan times (down to a few seconds for a whole XCT scan using a polychromatic beam).

The three-stripe design of the DMM (in addition to the use of the available DCM) allows a flexible shaping of the spectrum with energy resolutions in three (or even four) different regimes: (0.01%), ∼1.5%, ∼4% and pink beam.

To fully exploit the higher beam flux, the detector was upgraded to a white-beam configuration (moving the first lens out of the transmitted beam) in combination with an sCMOS camera. The new system supports recording of up to 100 full fps.

The installation of a slip ring into the turntable completes the upgrade and allows continuous rotation of the stage. The slip ring not only solves the problem of cable winding during XCT scans but also allows routing of cables to the sample to accommodate sample environment control.

The new capabilities for faster and more flexible XCT are numerous. We have demonstrated the versatility of the new BAMline using several scan modes on a pouch Li battery. Currently planned experiments include the use of an *in situ* tension–compression–torsion load frame, the *in situ* use of a furnace and the *in situ* use of a potentiostat, to cycle batteries. Trivially, *ex situ* experiments will also profit from the upgrade. As the scan duration is sensibly lowered, the number of samples can be increased, thereby allowing much larger sample throughput (*e.g.* to investigate in more detail the effect of manufacturing/processing parameter on the defect distribution in additively manufactured materials).

Shorter acquisition times also lead to higher data rates and bring the necessity of faster, but still thorough, data handling. Therefore, much effort is also put into the available on-site processing hardware and software solutions. The upgrades to the data-handling and reconstruction capabilities will be reported in a companion article.

## Figures and Tables

**Table:** 

Pd layer (total reflection)					
Energy range (keV)	∼18–22	∼15–25	∼20–30	∼20–40	∼30–40
Vertical beam size (mm)	1.8	1.5	1.3	1.0	1.0
Incident angle θ (°)	0.17	0.14	0.12	0.095	0.095
Filter (µm)	1000 Al + 50 Cu	700 Al	200 Al + 50 Cu	200 Al + 50 Cu	250 Cu
Flux peak (photons s^−1^ 0.1%bw)	∼1.8 × 10^9^	∼1 × 10^10^	∼6 × 10^9^	∼6 × 10^9^	∼1.6 × 10^9^

**Table:** 

W/Si DMM (Bragg diffraction)								
*E* (keV)	8	10	15	20	30	40	50	60
Vertical beam size (mm)	14.8	11.8	7.9	5.9	3.9	3.0	2.4	2.0
Incident angle θ (°)	1.393	1.113	0.743	0.558	0.373	0.280	0.224	0.187
FWHM (eV)	288	325	528	788	1292	1774	2243	2693
Δ*E*/*E* (%)	3.6	3.3	3.5	3.9	4.3	4.4	4.5	4.5

**Table:** 

Mo/B_4_C DMM (Bragg diffraction)								
*E* (keV)	8	10	15	20	30	40	50	60
Vertical beam size (mm)	16.8	13.5	9.0	6.7	4.5	3.3	2.7	2.2
Incident angle θ (°)	1.580	1.270	0.843	0.630	0.421	0.316	0.253	0.211
FWHM (eV)	124	164	257	270	495	701	898	1092
Δ*E*/*E* (%)	1.6	1.6	1.7	1.4	1.7	1.8	1.8	1.8

**Table 2:** Available lenses, pixel sizes, fields of view and scintillator thicknesses

White-beam microscope setup (Optique-Peter)			sCMOS camera 2560 × 2160 pixel	CdWO_4_ scintillator
Lens	NA	Field of view (mm)	Effective pixel size (µm)	Thickness (µm)
WB 2×	0.055	9.2 × 7.8	3.61	150
WB 5×	0.14	3.7 × 3.1	1.44	60
WB 10×	0.28	1.8 × 1.5	0.72	60
WB 20×	0.42	0.92 × 0.78	0.36	60
